# Fibroma of tendon sheath in planta

**DOI:** 10.1186/s40064-016-2260-z

**Published:** 2016-05-10

**Authors:** Hui Lu, Qiang Chen, Hui Shen, Xiang-qian Shen, Shou-cheng Wu

**Affiliations:** Department of Hand Surgery, The First Affiliated Hospital, College of Medicine, Zhejiang University, #79 Qingchun Road, Hangzhou, 310003 Zhejiang Province People’s Republic of China

**Keywords:** Fibroma of tendon sheath, Planta, Neurovascular bundle involvement

## Abstract

**Purpose:**

Fibroma of tendon sheath in planta is comparatively rare, and its differentiate diagnose, tumour features, treatment and complications were lack of retrospective study in clinics.

**Methods:**

This was a retrospective study of 13 patients (seven women, six men) operated between July 2001 and May 2013 for FTS in planta. The average age at the time of the procedure was 49.8 ± 8.3 years old (range 31–64). The female-to-male ratio was 9:4. Before the surgery, anteroposterior, lateral and oblique position of foot X-rays were performed in all patients. Ultrasonography (n = 11) and magnetic resonance imaging (n = 11) were performed selectively. The tumor located on the metatarsal par (n = 6), the central part of plantar (n = 4), the lateral part (n = 2) and the medial part (n = 1). Eight patients presented with painless mass (62 % of cases), while five patients presented with pain mass (38 % of cases). No patient had bony erosion. This paper studies the different features of FTS and classifies them into two types—superficial type that tumour grows at planter fascia; deep type that breaks through the planter fascia growing around tendon and joint capsule. Eight and five patients were diagnosed as superficial type and deep type respectively.

**Results:**

In all cases, the tumor was excised, pathological results was FTS. The mean follow-up period was 3.2 ± 1.1 years (range 2–7) years. Five patients had neurovascular bundle involvement (38 % of cases). Two patients had a recurrence (15 % of cases), they undergone another operation. Four patients had a pain (31 % of cases), two patients had numbness (15 % of cases), and one patient had pain and numbness (8 % of cases). They recovered after conservative treatment.

**Conclusion:**

For the FTS that grows in the plantar, we should select differential diagnosis and the corresponding therapy according to the features of two types, also the prognosis is different.

**Level of evidence:**

IV.

## Background

In 1936, Gechickter and Copeland (Chung and Enzinger 1979) first described a benign soft tissue lesion as FTS. FTS typically shows a small, well-circumscribed subcutaneous tumour. FTS can occur at any age, with a peak incidence at 20–50 years of age (Chung and Enzinger 1979). Chung and Enzinger (1979) reported 138 cases, in their series, 98 % of the lesions were in the extremities, and 82 % were in the upper extremities. Five cases occurred in the foot, and four of them demonstrated that the tumor was located in the plantar region. Excision has been the treatment of choice. FTS originates in the tendon or tendon sheath (Chung and Enzinger 1979; Fox et al. 2003). A high incidence of local recurrence, ranging from 11 to 25 % has previously been reported (Chung and Enzinger 1979; Humphreys et al. 1986). FTS in planta is comparatively rare, can be hard to distinguish from other lesions, only isolated case reports in previous literature. We report its clinical presentation, differentiate diagnose, treatment, tumour features and outcome in a retrospective study,which can be used to guide treatment in clinic.

## Patients and methods

From the First Affiliated Hospital, College of Medicine, Zhejiang University. This was a retrospective study with 13 patients (seven women, six men) presenting with a FTS in planta and operated between July 2001 and May 2013. Ethical approval was given by the medical ethics committee of the First Affiliated Hospital, College of Medicine, Zhejiang University. The average age was 49.8 ± 8.3 years (range 31–64). All the patients presented with gradually progressive swelling mass. Eight patients presented with painless mass (62 % of cases), while five patients presented with pain (38 % of cases). An injury to the area had preceded the appearance of the lesion (n = 4). Before the operations, anteroposterior, lateral and oblique position of foot X-rays were performed in all patients. Ultrasonography (n = 11) and magnetic resonance imaging (MRI) (n = 11) were performed selectively. The lesions were located on the metatarsal par (n = 6), the central part of plantar (n = 4), the lateral part (n = 2) and the medial part (n = 1) (Table 1). No patient had multiple lesions. All patients received fine needle biopsy. According to the different locations and features of tumours, we try to divides the tumours into two types (Table 2)—superficial type that the mass grows at planter fascia, and deep type that breaks through the planter fascia and grows around tendon and joint capsule. All patients underwent surgery to remove the tumour by an independent Surgeon. The surgical procedure was performed under regional or general anesthesia. A brachial tourniquet was used systematically and the procedure was carried out with surgical loupes. After the surgery, the drainage tube needed placing and no antibiotics would be used. All patients were able to walk with load under the protection of braces when the incision had healed. All patients would be evaluated again by an independent Surgeon. A clinical questionnaire was completed over the telephone and at the clinic. All the patients had been interviewed and completed regular follow-up visits. The clinical and radiographic examinations would be performed regularly in the follow-up study. Three patients (23 % of cases) were undergone the rechecks of X-ray, Ultrasonography and MRI were used as a tumor recurrence standard.

**Table 1:** Basic clinical data

Case	Age	Sex	Location	Previous injury	Follow-up period	Neurovascular involvement
1	56	Female	Metatarsal	No	3	Yes
2	31	Male	Metatarsal	No	7	No
3	46	Female	Central	Yes	4	No
4	57	Female	Medial	No	3	No
5	64	Female	Central	Yes	5	No
6	43	Female	Lateral	No	2	No
7	42	Male	Central	No	2	No
8	50	Female	Metatarsal	No	4	Yes
9	63	Female	Lateral	No	3	No
10	34	Male	Metatarsal	Yes	2	No
11	55	Female	Metatarsal	No	3	Yes
12	49	Male	Central	No	2	Yes
13	58	Female	Metatarsal	Yes	2	Yes

**Table 2:** Different types with different results

Case	Type	Neurovascular involvement	Results	Sequelae
1	Deep	Yes	R	NP
2	Superficial	No	NSR	N/A
3	Superficial	No	NSR	P
4	Superficial	No	NSR	N/A
5	Superficial	No	NSR	N/A
6	Superficial	No	NSR	P
7	Superficial	No	NSR	N/A
8	Deep	Yes	NSR	N
9	Superficial	No	NSR	P
10	Deep	No	NSR	N/A
11	Deep	Yes	R	N
12	Superficial	Yes	NSR	P
13	Deep	Yes	NSR	N/A

## Results

Pathological results confirmed it as FTS, which composed of tightly packed spindle cells surrounded by collagen fibers. The mean follow-up period was 3.2 ± 1.1 years (range 2–7). No patient had bony erosion. Eight and five patients were diagnosed as superficial type and deep type respectively. Five patients had neurovascular bundle involvement (38 % of cases) with decompression in operation. Clinical suspicion of neurovascular bundle involvement arose when the patients presented with pricking or irradiated pain. Complications have been recorded including tumour recurrence pain and numbness. Two patients had a recurrence (15 % of cases), they undergone reoperation. Four patients had a pain (31 % of cases), two patients had numbness (15 % of cases), and one patients had pain and numbness (8 % of cases). Conservative management with Neurotrophy medicine and NSIAD were appropriate for those patients.

## Discussion

**Fig. 1:**
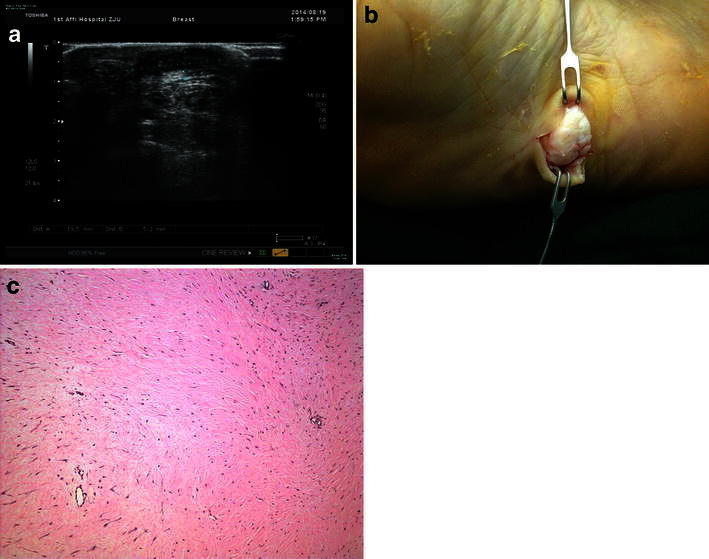
**a** Ultrasound indicates the lesions were located under superficial layer of the planta before operation. **b** The tumor was located above the plantar fascia. **c** For the superficial type, the vogue lobulated lesion was arranged. The lesion constituted discrete fibroblast, the confined lacuna vasorum and red collagen fibers. Fibroblast had the shape of short shuttle, the partial cells were rich with bundles of spindle-shaped arrangement (magnification ×100)

**Fig. 2:**
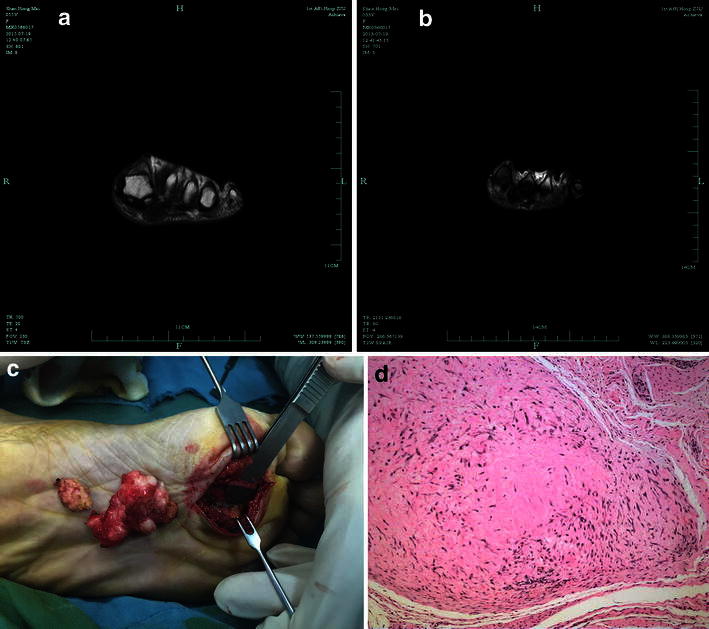
**a** The tumor was low signal intensity on T1-weighted images, located in the distal planta before the operation. **b** The tumor was low signal intensity on T2-weighted images before the operation. **c** The tumor was adhered with the common plantar digital nerve. **d** For the deep type, the lesion had a lobulated appearance. Every lobulated one was separated with the narrowed and lacunar infarction, around which the remaining tendon sheath structure could be seen. It constituted discrete fibroblast, the confined lacuna vasorum and red collagen fibers. The fibroblast had the shape of short shuttle, the partial cells were rich with bundles of spindle-shaped arrangement (original magnification ×100)

The diagnosis of FTS is supposed to be confirmed by several aspects such as the patient’s history, the clinical examination, and radiologic and pathologic findings. The major differential diagnosis of superficial type of FTS (Fig. 1) includes plantar fasciitis, plantar fascial fibromatosis and nodular fasciitis. Plantar fasciitis occurs because the supporting plantar fascia of the arch becomes strained and inflamed. The prevalence of plantar fasciitis in the general population is estimated to range from 3.6 to 7 % (Dunn et al. 2004; Hill et al. 2008), is a common problem that affects sport participants as well as inactive middle-aged individuals (Davis et al. 1994; Martin et al. 1998). The patients diagnosed as the superficial type had trauma history commonly. Even if some of them did not have trauma history, the tumor occurring resulted from surface of planta was rubbed frequently. Plantar fascial fibromatosis (Bedogni and Dal Monte 1962; Koudela et al. 2010; Dartoy et al. 1990), also known as Ledderhose’s disease, is a relatively uncommon non-malignant thickening of the feet’s deep connective fascia. At the beginning of the disease, cords and nodules start growing along the plantar fascia. Therefore, that is easy to be confused with FTS. The nodules are slowly growing, most of them are often found in the central and medial portions of the plantar fascia. And at the end of the disease, the cords thicken, the toes stiffen and bend (Barnes et al. 2009). However, the FTS on the surface of plantar cannot influence on the toes. Nodular fasciitis is a benign, fibroproliferative lesion that is thought to represent a reaction to injury or inflammation (Cotter et al. 2000). It most frequently affects the upper extremities, especially the volar aspect of the forearm (Brown and Carty 2005). The characteristic vascular pattern of FTS is not seen in nodular fasciitis microscopically (Hornick and Fletcher 2006). Pathologic examination is the gold standard for diagnosis of FTS, sometimes diagnosis requires immunohistochemical confirmation. Deep type of FTS (Fig. 2) should be differentiated from giant cell tumor of the tendon sheath (GCTTS). These two lesions are similar in size, location, and gross appearance. GCTTS mostly occurs in the toes (Gibbons et al. 2002; Darwish and Haddad 2008). Satti (1992) suggested the pathological study between FTS and GCTTS is difficult to identify. GCTTS may present with bone erosion or destruction in radiography (Uriburu and Levy 1998; Lu et al. 2015). There were infrequent above imaging features in FTS. And there were no giant cells in FTS observed under microscope.

Both Ultrasound and MRI are options for FTS. We choose ultrasound for superficial type of FTS. Ultrasound is an important way with its advantage of noninvasive convenience, also making a more definite diagnosis. Solid soft tissue tumors may be confused with cystic masses on ultrasonography (Lee et al. 2010), so we suggest that using MRI as an additional screening tool. Normally, FTS has a lower signal or slightly higher intensity signal on T2-weighted images. But due to the edema in the tumour or capillary vascularity surrounding tumour reacts high intensity signal on T2-weighted images (Fox et al. 2003). Deep type of FTS especially when neurovascular was involved, MRI play an important role in studying the anatomy, and avoid nerves damage during surgery. The images of tendon and tendon sheath provided effective information for resection range and selecting the most reasonable incision. MRI also would help the clinician finding early recurrence more effectively.

The treatment of FTS is surgical resection. FTS is excised together with the overlying tendon sheath. The indication for surgery is aches and pain. But patients had FTS without any painful discomfort initially also need early resection. Excise to relieve symptoms but preserve function, may be difficult to remove from adherent tendons. Preoperative diagnosis and type are helpful for the design of the operation planning. Superficial type of FTS was easier to remove. But deep type of FTS grows around tendon and joint capsule, especially some patients had neurovascular bundle involved, is difficult to remove from adherent tendons. The tumor cannot be excised completely, which is the main reason to have a recurrence after the surgery. The nerve injury should be avoided during operation. Nerve injury during operation could lead to numbness. Painful scar is also a difficult problem. Light therapy and lasers are meant to minimize its appearance. A malignant transformation has never been described. Only tumour recurrence needs reoperation in follow-up.

## Conclusion

According to the different types of FTS growing in planta, the prognosis is different. Preoperative definite type is helpful for the design of the operation plan. The deep type commonly has adhesion to the neurovascular bundle. It is difficult to excise completely, has a high recurrence after surgery as well as easy to produce complications of numbness and pain. MRI is developed in an attempt to minimize surgical damage and preserve nerves function.

## Abbreviations

FTS: fibroma of tendon sheath
GCTTS: giant cell tumor of the tendon sheath
MRI: magnetic resonance imaging
